# Antifungal Resistance Profile, Biofilm Formation, and Virulence Factor Production in Candida krusei Isolates From HIV-Infected Patients in Cameroon

**DOI:** 10.7759/cureus.44213

**Published:** 2023-08-27

**Authors:** Cyrille Levis Kountchou, Michel Noubom, Borel Ndezo Bisso, Thierry Ngouana Kammalac, Alfred Itor Ekpo, Aude Ngueguim Dougue, Claude Nangwat, Martin Oyono, Stéphane Ranque, Jean Paul Dzoyem

**Affiliations:** 1 Department of Biochemistry, University of Dschang, Dschang, CMR; 2 Department of Microbiology, Hematology and Immunology, Faculty of Medicine and Pharmaceutical Sciences, University of Dschang, Dschang, CMR; 3 Laboratoire Sion, Unité de Recherche Biomédicale, Yaounde, CMR; 4 Department of Biochemistry and Molecular Biology, University of Buea, Buea, CMR; 5 Department of Biochemistry, Faculty of Science, University of Yaoundé I, Yaoundé, CMR; 6 Laboratoire de Biologie Humaine, Institute of Medical Research and Medicinal Plants Studies, Yaounde, CMR; 7 Department of Infectious Diseases, Assistance Publique Hôpitaux de Marseille, Vecteurs et Infections Tropicales et Méditerranéennes, Aix-Marseille Université, Marseille, FRA; 8 Department of Infectious Diseases, Institut de Recherche pour le Développement, Marseille, FRA

**Keywords:** human immunodeficiency virus, hiv, proteinase, phospholipase, virulence factors, biofilm, resistance, antifungal, candida krusei

## Abstract

Background

Fungal infections mainly caused by *Candida krusei *are increasing rapidly and represent a serious public health problem in human immunodeficiency virus (HIV)-infected patients. This study aimed to investigate the antifungal susceptibility profile and virulence factors in *C. krusei* isolated from HIV-infected patients.

Methodology

Isolates were identified by biochemical and molecular methods. The antifungal resistance profile was established based on the antifungal susceptibility test performed using the Sensititre YeastOne™ (Thermo Fisher Scientific, Waltham, MA) microdilution technique. The production of phospholipase and proteinase was detected by standard methods. Biofilm formation was performed by the microtiter plate method.

Results

A total of 73 isolates of *C. krusei* were recovered from stool, oral swabs, vaginal swabs, and urine samples. The highest number of *C. krusei *isolates (49, 67.05%)was recovered from stool samples. A total of 32.56% of the *C. krusei* isolates were multidrug-resistant (MDR). The patients living with HIV and not receiving antiretroviral treatment displayed the highest number of *C. krusei *isolates (29, 39.76%), whereas the patients living with HIV on antiretroviral therapy exhibited the lowest number of *C. krusei *isolates (2, 2.72%). All isolates were categorized as strong biofilm producers. Among the production of hydrolytic enzymes, 25 (58.13%) and 24 (55.81%) of *C. krusei* isolates were classified as strong phospholipase and proteinase producers, respectively.

Conclusion

The *C. krusei* isolates obtained in this study were MDR and strongly expressed biofilm formation and both phospholipase and proteinase hydrolytic enzymes. The results show how pathogenic *C. krusei* is in the HIV-infected population and will contribute toward the management of* C.* *krusei-*related infections, which may help improve the life quality of people living with HIV.

## Introduction

Human immunodeficiency virus (HIV), which causes acquired immunodeficiency syndrome (AIDS), has caused significant devastation worldwide. Approximately 38.4 million people are currently living with HIV, and 650,000 HIV deaths occurred in 2021 [1]. Furthermore, AIDS remains a major public health issue in all countries in the world, with a more significant impact being seen in developing countries [2]. The progressive damage HIV infection causes to the host immune system opens the door to opportunistic infections such as fungal infections. Among these fungal infections, candidiasis is commonly encountered in HIV-infected patients [3], and the increase of such patients has increased the occurrence of opportunistic infection due to *Candida* species [4]. Several studies have reported that even with the use of highly active antiretroviral therapy (HAART) in HIV-infected patients, opportunistic infections represent a serious health problem [3]. Approximately 60-80% of HIV-infected patients develop candidiasis [5]. In developing countries, the mortality rate due to HIV infection leading to AIDS was four to 15 times higher than that of developing countries, although systematic data are lacking [6]. Among the different *Candida* species, *Candida albicans* is the main cause of candidiasis. However, in recent years, significant cases of candidiasis have been attributed to non-*albicans Candida* species, including *Candida krusei* [4]. *C. krusei* represents an important cause of invasive candidiasis associated with a high mortality rate (30-60%) [7]. In HIV-infected patients, the morbidity and mortality rates are particularly high [8]. However, despite the availability of an antifungal therapeutic arsenal, *C. krusei* infections remain a serious public health problem owing to its decreased susceptibility to a range of antifungals and increasing intrinsic fluconazole resistance [9].

Moreover, the invasive capacity of *C. krusei *depends on the production of several virulence factors such as hemolysin production, biofilm formation, and the production of phospholipase and proteinase [8]. Biofilms are structured microbial communities that attach to biotic or abiotic surfaces and are enclosed in an extracellular matrix composed of water, polysaccharides, proteins, lipids, and extracellular DNA [10]. The ability of microorganisms to form biofilms provides them with a protective environment against both the host immune system and antibiotics, which may promote their persistence, tissue invasion, and destruction [11]. Phospholipase and proteinase are hydrolytic enzymes that cause tissue damage and the dissemination of infections [12]. The phospholipase hydrolyzes host cell membrane phospholipids, leading to membrane damage, whereas proteinase hydrolyzes the peptide bonds of proteins, which contributes to microbial adhesion and invasion of cells [13]. Knowledge of these virulence factors can be an important tool for understanding the pathogenesis of candidiasis caused by *C. krusei*. In addition, the isolation, identification, and antifungal susceptibility testing of *C. krusei* have paramount significance in the management of fungal infections caused by *C. krusei*. However, little is known about the ability of isolates of *C. krusei* species isolated from HIV-infected patients to produce virulence factors. Moreover, little information is available on the antifungal susceptibility profile of *C. krusei* isolated from HIV-infected patients. Therefore, this study aimed to investigate the virulence factors and antifungal resistance patterns of *C. krusei* isolated from HIV-infected patients.

## Materials and methods

Study design

A cross-sectional study was conducted at Yaoundé Central Hospital and Bafoussam Regional Hospital for a period of two years from October 2018 to December 2020. The study protocol was approved by the Cameroon National Ethics Committee for Human Health Research (CE N°22583/CRERSHC/21). Patients of both genders, age 21 years and above, with or without symptoms of fungal infections, and who provided informed consent were included in the study. We excluded patients on antifungal drug therapy in the three months before the study and with underlying medical conditions other than HIV infection that favor candidiasis.

Sample collection and identification of isolates

From 804 patients recruited, 2,754 clinical samples (stool, urine, and vaginal and oral swabs) were collected. Each sample was inoculated on Sabouraud dextrose agar supplemented with chloramphenicol for 48 hours at 37°C. Primary identification was performed phenotypically by subculture on CHROMagar Candida medium (CHROMagar from bioMérieux, Marcy l’Etoile, France). Then, a second identification was performed by matrix-assisted laser desorption/ionization-time of flight (MALDI-TOF) mass spectrometry (MS), as described by Cassagne et al. [14]. Briefly, analyses were performed on a Microflex LT (Bruker Daltonics GmbH, Bremen, Germany) equipped with a nitrogen laser (337 nm). The mass ranges from 2,000 to 20,000 Da were recorded by using the linear mode. *C. krusei *ATCC (American Type Culture Collection) 14243 was used for quality control. The spectra were compared to the MALDI Biotyper v3.0 software (Bruker Daltonics GmbH) containing the Bruker Daltonics database supplemented with our in-house yeast reference spectra for identification [14].

Determination of the antifungal resistance profile

Antifungal susceptibility testing was carried out by Sensititre YeastOne™ (Thermo Fisher Scientific, Waltham, MA) according to the manufacturer’s instructions using a 0.5 McFarland inoculum from overnight cultures followed by incubation at 35ºC for 48 hours. The antifungal drugs tested were fluconazole, amphotericin B, and 5-flucytosine. The lowest concentration of antifungal drug that inhibited 100% of the yeast growth was defined as the minimum inhibitory concentration (MIC). *C. krusei *ATCC™ 14243 and *C. parapsilosis* ATCC™ 22019 were used for quality control. Results were interpreted using clinical breakpoints (CBPs) following the Clinical and Laboratory Standards Institute (CLSI) M60 1st edition 2017 guidelines (CLSI, 2017) and epidemiological cut-off values (ECVs) following CLSI M59 2nd edition 2018 guidelines (CLSI, 2018) [15,16]. The isolates were categorized into distinct resistotypes as previously described [17]. A multidrug-resistant (MDR) isolate was defined as an isolate showing resistance to three or more classes of antifungals tested. A multiple antifungal resistance (MAR) index was calculated as follows: MAR = number of isolate resistance to antifungal/total number of antifungals used. A MAR > 0.2 indicates a high-risk source of contamination [18,19].

Biofilm formation assay

The biofilm formation assay was performed by the microtiter plate method as described by Bisso et al. [10], with slight modifications. In brief, the wells of a 96-well flat-bottomed polystyrene plate were filled with 100 μL of fungal inoculum (1.5 x 10^6^ CFU/mL) and 100 μL of Sabouraud dextrose broth (SDB) supplemented with 5% glucose. Then, the microplate was incubated for 48 hours at 37ºC on a shaker (120 rev/minute). After the incubation time, the medium was removed, and the microplate was washed three times with sterile ultrapure water to remove the planktonic cells. The microplate was dried at room temperature for 30 minutes to fix the adherent cells. After incubation, the adherent cells were stained with 200 μL of crystal violet (1%), and the microplate was incubated at room temperature for 20 minutes. After the incubation period, the excess crystal violet was removed, and the dye bound to biofilm cells was solubilized with 200 μL of 33% acetic acid. Wells containing SDB supplemented with 5% glucose and the *C. krusei* ATCC 14243 strain were used as blank and positive controls, respectively. The absorbance of the microplate was measured at 570 nm using a microplate reader (Infinite M200, Tecan, Männedorf, Switzerland). The isolates that formed biofilms with optical density (OD) values higher than that of the positive control were considered strong biofilm producers, whereas those isolates with OD values less than that of the positive control were considered weak biofilm producers.

Phospholipase production

The *C. krusei* isolates were screened for phospholipase production using the egg yolk agar plate method described by Hekmatpanah et al. [20]. Briefly, 10 µL of each fungal inoculum (1.5 x 10^6^ CFU/mL) was spotted on the egg yolk agar plate. After five days of incubation at 37ºC, the diameter of the colony and precipitation around the colony were measured. Phospholipase production (Pz) was expressed as follows: Pz = colony diameter/colony diameter + precipitation zone. The degree of Pz was categorized as follows: negative (Pz = 1), weak (0.80 < Pz < 0.99), moderate (0.70 < Pz < 0.79), and strong (Pz < 0.70) [20].

Proteinase production

The bovine serum albumin (BSA) agar method described by Hekmatpanah et al. [20] was used to detect proteinase production in *C. krusei* isolates. The medium included BSA (0.2%), yeast extract (0.01%), glucose (1.17%), and agar (2%). A volume of 10 µL of each fungal inoculum (1.5 x 10^6^ CFU/mL) was spotted onto plates and then incubated for five days at 37ºC. The proteinase production was detected by the formation of a transparent halo around the yeast colonies. Proteinase production (Prz) was expressed and scored as described above for phospholipase production.

Statistical analysis

GraphPad Prism 8 software (GraphPad Software, San Diego, CA) was used for statistical analysis. The results are shown as the mean ± standard deviation of three independent experiments. The statistically significant differences between experimental groups were evaluated by two-way analysis of variance with a Dunnett’s test for multiple comparisons. *p < 0.05, **p < 0.01, ***p < 0.001, and ****p < 0.0001 were considered statistically significant.

## Results

Distribution of *C. krusei* isolates

Table 1 shows the distribution of *C. krusei *isolates according to sex, age, sample type, CD4 count, HAART status, and locality. Out of 2,754 samples collected, a total of 73 isolates of *C. krusei* were identified, of which 43 (58.9%) were classified as pathogenic isolates based on the criteria described by Bouchara et al. [21]. Female patients had a higher number of *C. krusei* isolates (59, 80.89%) compared to male patients (14, 19.10%). The age group of 31-40 years accounted for the highest number of *C. krusei* isolates (39, 53.41%), followed by 21-30 years (12, 16.37%), 51-60 years (10, 13.64%), and 41-50 years (9, 12.47%), whereas the age group ≥ 61 years had the lowest number (3, 4.09%). The highest number of *C. krusei *isolates was obtained from stool (49, 67.05%) followed by oral swabs (10, 13.64%) and vaginal swabs (9, 12.47%), whereas urine samples had the lowest number (5, 6.82%). Concerning patients’ CD4 count, the highest number of *C. krusei* isolates (29, 39.73%) was obtained in patients with a CD4 count of 200-349 cells/mm^3^, whereas patients with a CD4 count greater than 200 cells/mm^3^ showed the lowest number (12, 16.44%). Among the HAART therapy status, patients living with HIV and not receiving treatment displayed the highest number of *C. krusei *isolates (71, 97.26%), whereas patients living with HIV on HAART therapy exhibited the lowest number (2, 2.74%). Among the localities, 51 (68.49%) and 22 (31.51%) isolates of *C. krusei* were obtained in Yaoundé Central Hospital and Bafoussam Regional Hospital, respectively. Of the 73 isolates identified, 43 isolates classified as pathogenic were selected for further study of their antifungal resistance profile and virulence factor production.

**Table 1:** Distribution of C. krusei isolates according to gender, age, sample type, CD4 count, HAART status, and locality BRH = Bafoussam Regional Hospital; YCH = Yaoundé Central Hospital; HAART = highly active antiretroviral therapy; HAART (+) = patients receiving highly active antiretroviral therapy; HAART (−) = patients not receiving highly active antiretroviral therapy.

Parameter	Category	Number of *C. krusei* isolates (N = 73)	Frequency (%)	Chi-square (X^2^)	p-value
Gender	Male	14	19.10	0.057	0.8064
	Female	59	80.89		
Age range (years)	21–30	12	16.37	6.428	0.0987
	31–40	39	53.41		
	41–50	9	12.47		
	51–60	10	13.64		
	≥61	3	4.09		
Sample type	Oral swab	10	13.64	4.066	0.3942
	Vaginal swab	9	12.47		
	Stool	49	67.05		
	Urine	5	6.82		
CD4 count (CD4/mm^3^)	<200	12	16.44	1.688	0.6870
	200–349	29	39.73		
	350–499	17	23.29		
	≥500	15	20.54		
HAART therapy status	HAART (+)	2	2.74	14.261	0.0002
	HAART (−)	71	97.26		
Locality of sample collection	BRH	22	31.51	7.524	0.113
	YCH	51	68.49		

Antifungal resistance profile

The antifungal resistance patterns (resistotypes) and resistance rate of *C. krusei* isolates are shown in Table 2. Resistotypes III, IV, and V showed MAR index values > 0.2 with six (13.95%), 18 (41.86%), and 14 (32.56%) *C. krusei* isolates, respectively. Additionally, resistotype III and IV were classified as non-MDR, whereas resistotype V was classified as MDR.

**Table 2:** Resistotype and resistance rate of C. krusei isolates FL = fluconazole; AMB = amphotericin B; 5-FC = 5-flucytosine; MAR = multiple antifungal resistance; MDR = multidrug resistant.

Resistotype	Antifungals	MAR index	Ratio of isolate, % (n)	MDR status
I	FL	0.111	6.98 (3)	Non-MDR
II	AMB	0.111	4.65 (2)	
III	AMB, FL	0.222	13.95 (6)	
IV	5-FC, FL	0.222	41.86 (18)	
V	5-FC, FL, AMB	0.333	32.56 (14)	MDR

Biofilm formation

**Figure 1:**
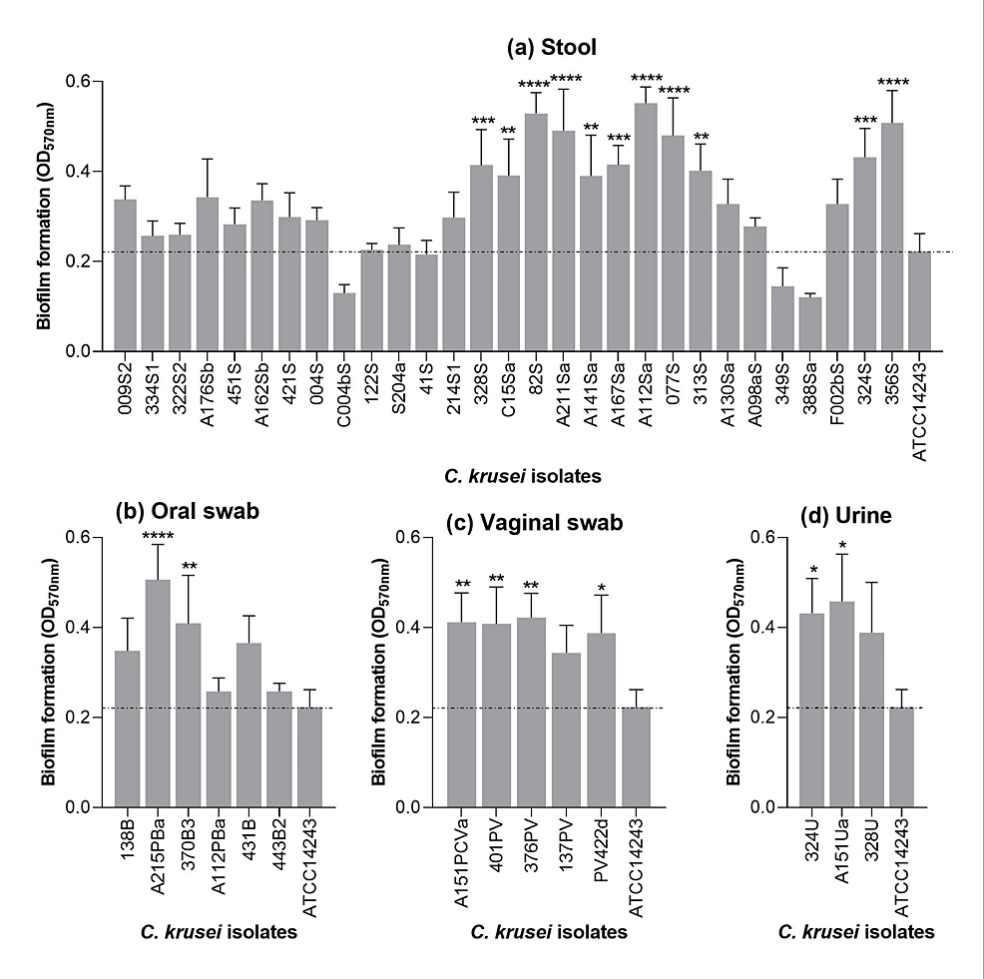
Biofilm formation by C. krusei isolates obtained from stool (a), oral swab (b), vaginal swab (c), and urine (d) samples The reference strain *C. krusei* ATCC 14243 used as a control was included in each graph.

The biofilm formation in 43 *C. krusei* isolates is plotted in Figure 1. Among isolates from stool, 25 (86.21%) were classified as strong biofilm producers, whereas four (13.79%) were categorized as weak biofilm producers. All isolates from the oral swab, vaginal swab, and urine samples were categorized as strong biofilm producers.

Phospholipase and proteinase production in *C. krusei* isolates

The production of hydrolytic enzymes was detected in *C. krusei* isolates, as shown in Table 3. For phospholipase, 25 (58.13%) isolates were strong producers, eight (18.60%) were moderate producers, six (13.95%) were weak producers, and four (9.30%) were non-producers. Regarding proteinase production, 24 (55.81%) isolates were strong producers, 10 (23.25%) were moderate producers, and nine (20.93%) were nonproducers.

**Table 3:** Production of phospholipase and proteinase in C. krusei isolates n = number of *C. krusei* isolates; % = frequency.

Virulence factors	Categorization			
	Non-production, n (%)	Weak, n (%)	Moderate, n (%)	Strong, n (%)
Phospholipase	4 (9.30)	6 (13.95)	8 (18.60)	25 (58.13)
Proteinase	9 (20.93)	0 (0.00)	10 (23.25)	24 (55.81)

## Discussion

Candidiasis is one of the most common opportunistic fungal infections and continues to be the cause of high morbidity and mortality rates, especially in HIV-positive patients [2]. Although *C. albicans* is the main species causing candidiasis, in recent years, a significant number of candidiasis cases have been attributed to non-*albicans Candida* species such as *C. krusei* [4]. In this study, a total of 73 *C. krusei* isolates were recovered from 2,754 clinical samples, giving a prevalence of 2.65%. A previous work reported that *C. krusei* were recovered at a higher frequency (18.1%) in HIV-infected patients [22]. The low frequency obtained in our study could be attributed to an increased use of HAART, which improves the immune system of patients. In the present study, a high frequency of *C. krusei* was reported in female patients (80.89%) compared to male patients (10.21%). This difference may be attributed to anatomical differences such as short urethra and proximity to the anus in females, and exposure to contamination among female and male populations [23,24]. Our results showed that *C. krusei* was most frequently isolated from stool samples in HIV-positive patients. The high frequency of *C. krusei* in stool samples could be attributed to the commensal nature of the yeast in the gastrointestinal tract. Moreover, HIV infection destroys the host immune system, leading to an increased risk of gastrointestinal infection [22] by fungi such as *Candida*. We observed a high prevalence of *C. krusei* isolates in patients living with HIV and not receiving HAART treatment (97.26%) compared to patients living with HIV and receiving HAART therapy (2.74%). This could justify the significantly decreased incidence of candidiasis in HIV-infected patients since the discovery of HAART therapy [25].

Treatment of fungal infections depends on the infected site, the host immunological status of the patient, and the yeast species isolated [26]. In our study, 32.56% of *C. krusei* isolates were MDR. This MDR could be explained by the scarcity of antifungal drug classes associated with intrinsic resistance and fungal species' ability to adapt under antifungal stress [27]. *C. krusei* has multiple virulence factors, including biofilm formation and secretion of hydrolytic enzymes. The adherence of yeast cells to host tissues through biofilm formation initiates colonization and provides a protective environment for microorganisms against both the host immune system and antibiotics [11,28]. Phospholipase breaks down phospholipids in the cell membrane of the epithelial cells, leading to cell membrane damage and lysis. Proteinase damages the surface host proteins and degrades the locally protective immunoglobulin A (IgA) and complement components 3 (C3) by hydrolysis of peptide bonds [28,29]. In this regard, the biofilm formation and two hydrolytic enzymes, including phospholipase and proteinase, were detected in *C. krusei* isolates. Our results showed that strong biofilm producers were observed in most *C. krusei* isolates recovered from different clinical samples. Additionally, most *C. krusei* isolates were strong phospholipase and proteinase producers (58.13% and 55.81%, respectively). The high expression of these virulence factors in *C. krusei *isolates could reflect their more virulent character compared to the isolates that more weakly produce these virulence factors. These results corroborate those of previous studies, which reported the high production of biofilm, phospholipase, and proteinase in non-*albicans Candida* species, including C*. krusei* [28,30].

Limitations of the study

The study was focused on HIV patients specifically in two healthcare centers located in two different regions of Cameroon; therefore, the data obtained cannot be applied to other regions or the whole country. Another limitation is the lack of longitudinal data. Since we conducted a cross-sectional study, the cross-sectional nature may have prevented the identification of potential changes in antifungal resistance, biofilm formation, and virulence factor production over time. We also faced limited resources issues in terms of funding for equipment, personnel, and some laboratory facilities, which had an impact on the quality and breadth of our study.

## Conclusions

*C. krusei* is an opportunistic pathogen involved in Candida infections in HIV-infected patients. Along with its intrinsic resistance to fluconazole, *C. krusei* infections are further complicated by the increasing prevalence of other triazoles and imidazole non-susceptibility in these strains. In addition to their propensity to form biofilms, the isolated *C. krusei* showed the ability to produce hydrolytic enzymes. The *C. krusei* isolates obtained in this study were MDR and strongly expressed biofilm formation and phospholipase and proteinase hydrolytic enzymes. These results add new insights into the contribution of virulence factors in the pathogenesis of *C. krusei* infection in HIV-infected patients. Our findings will contribute to a better understanding of the pathogenicity of *C. krusei* and the management of its infection, which may suggest new therapeutic strategies against candidiasis in people living with HIV infection.
